# Artificial intelligence applications in automated dental report generation – a scoping review

**DOI:** 10.3389/froh.2026.1817718

**Published:** 2026-06-02

**Authors:** Madeline Yon, Ethan Ng, Michael M. Bornstein

**Affiliations:** 1 Centre for Oral Clinical Research, Institute of Dentistry, Faculty of Medicine and Dentistry, Queen Mary University of London (QMUL), London, United Kingdom; 2 Department of Restorative Dentistry, National Dental Centre Singapore, Singapore, Singapore; 3 Department of Oral Health and Medicine, University Center for Dental Medicine Basel UZB, University of Basel, Basel, Switzerland

**Keywords:** artificial intelligence, dental, GPT, large language models, review

## Abstract

**Background:**

Artificial intelligence (AI), particularly large language models and natural language processing (NLP), has enabled the structured synthesis and summarisation of complex clinical data. In healthcare, AI-driven report generation has the potential to improve efficiency, reduce clinician workload, and enhance communication with patients and healthcare professionals. This scoping review aimed to map current developments in AI applications for dental report generation and to identify existing research gaps.

**Materials and methods:**

A systematic search of Embase, MEDLINE via Ovid, and IEEE Xplore was conducted for studies published between January 2015 and March 2026. Eligible studies described development, evaluation, or application of AI systems for generating dental reports from imaging, textual, or voice inputs. Extracted data on AI models, input modality, language, and evaluation methods were summarised descriptively.

**Results:**

1,265 records were identified, of which seven studies met inclusion criteria. Six focused on radiology report generation from panoramic radiographs, and one generated clinical examination reports from voice-transcribed charting. NLP-based models, including GPT variants and fine-tuned large language models, were used to generate final reports. Performance was evaluated using metrics including ROUGE, BLEU, BERTScore, hallucination analysis, structural validity, response latency, output length, readability indices, clinician ratings, and patient questionnaires. AI-generated reports demonstrated high accuracy for common findings and readability comparable to human-authored reports. Simplified AI-adapted versions improved patient-rated clarity. However, heterogeneity in report types, datasets, languages, and evaluation metrics limited direct comparison.

**Conclusion:**

AI systems show promising capability in generating dental reports from diverse inputs and customisation for professional or non-professional audience. Standardised evaluation frameworks, larger multilingual datasets, and assessment of patient comprehension are needed before routine clinical implementation.

## 1 Introduction

The rapid advancement of artificial intelligence (AI), particularly the emergence of large language models (LLM) and natural language processing (NLP) systems, has transformed the way complex and unstructured textual data can be curated, analysed, and synthesised. These technologies enable the structured summarisation of heterogeneous clinical information, and the generation of coherent outputs tailored to different audiences, including healthcare professionals and patients. In medicine, AI-assisted documentation tools have demonstrated potential benefits such as rapid data extraction, automated structuring of clinical information, and preparation of patient letters and radiology reports (1–3). Similarly, dental documentation – including clinical notes, radiology reports, referral letters, and patient correspondence – serves a critical role in recording and communicating a patient's oral health status, diagnoses, risk assessments, and treatment plans. Established standards emphasise that records should be accurate, complete, legible, and contemporaneous (4, 5). Radiology reporting guidelines further recommend clear descriptions of findings, inclusion of differential diagnoses, and specific management recommendations (6).

More broadly, NLP has been shown to play a key role in extracting and organising clinical narratives from unstructured health records, facilitating improved interoperability and decision support system (7). In parallel, industry-driven solutions are emerging, including platforms such as DentScribe, ScanWise, Yalha, and Alegra, which aim to automate aspects of dental documentation, radiographic interpretation, and clinical reporting workflows. These systems highlight the growing translational potential of AI-driven report generation beyond research settings. However, despite these developments, there remains limited peer-reviewed evidence evaluating their performance, generalisability, and clinical utility, underscoring the need for systematic synthesis of available literature.

Recent developments in digital dentistry have seen AI integrated across multiple domains, including computer vision models for radiographic caries detection and bone loss (8–11), oral hygiene education (12), data extraction from electronic health records (1), implant treatment planning (13), and prompt engineering to provide dental education for patients (14) as well as students (15, 16). These applications are underpinned by advances in machine learning and deep learning, which have demonstrated high accuracy in diagnostic and predictive tasks across dental specialties (17). However, interoperability of such AI systems remains low. Most existing AI systems in dentistry remain task-specific, focusing on diagnostic or analytical functions, while few studies have integrated these functions or data modalities together for downstream communication processes such as report generation. Indeed, current NLP applications in dentistry are largely limited to information extraction from clinical notes, with relatively few studies addressing automated narrative generation (18).

Although generative AI systems have demonstrated capabilities in producing structured clinical summaries, such as discharge summaries, referral letters, and radiology reports, AI-driven dental report generation remains an underexplored area. Clinical documentation continues to represent a substantial administrative burden, occupying a significant proportion of clinicians’ time and contributing to workflow inefficiencies (19). Emerging developments such as multimodal AI systems, which integrate textual, imaging, and structured electronic health record data, may further expand the potential for automated report generation and clinical documentation. Through generative summarisation, tasks such as report generation, preparing discharge summaries and patient clinic letters could be performed (20, 21), while missing data imputation could enable the filling in of important details such as diagnoses based on electronic health records (20). Importantly, these tools may improve efficiency, enhance completeness of records, while ensuring that such documentation remains individualised, and reduce administrative burden – factors associated with clinician workload and burnout (22, 23).

Despite a growing interest in AI applications within dentistry, the specific domain of AI-driven dental report generation remains fragmented. Existing studies are limited in number and characterised by heterogeneous methodologies, inconsistent evaluation metrics, and variable reporting quality. This lack of standardisation limits comparability across studies and hinders translation into clinical practice. A comprehensive synthesis of current evidence is therefore warranted.

Therefore, the primary aim of this scoping review is to systematically map and characterise the existing literature on artificial intelligence applications in dental report generation, with a focus on the technologies employed and their contexts of use. The secondary aims are to identify research gaps, methodological limitations, and key challenges in this rapidly evolving field.

## 2 Materials and methods

This scoping review was reported following the Preferred Reporting Items for Systematic Reviews and Meta-Analyses (PRISMA) Extension for Scoping Reviews (PRISMA-ScR) Checklist (24). No protocol was pre-registered online for this review. The research questions that guided this review were:

1. What types of AI technologies have been applied in automated dental report generation?
2. In what clinical contexts have these systems been used?
3. What is the performance of these systems and what are their limitations?
4. What are the potential areas of research to further investigate the utility of AI automated dental report generation?

The Population-Concept-Context of the study was formulated following the Joanna Briggs Institute guidelines (25) (Table 1).

**Table 1:** Population-concept-context framework.

Domain	Category	Description
Population	Intended recipient	Dental professionals, including dentists and radiologists, and/or patients
	Type of report	Dental radiology reports, clinical notes, patient letters, summaries, imaging reports
	Clinical focus	Both general and dental specialties
Concept	AI task	Automated dental report generation
	AI methodology	Including natural language processing (NLP), visual-language models and large language models (LLM)
Context	Clinical setting	Routine clinical dental practice settings, including dental imaging

### 2.1 Eligibility criteria

Studies were eligible for inclusion if they included AI-assisted generation of dental documentation intended for communication with clinicians and/or patients, such as dental radiology reports, clinical notes, patient letters, summaries, and imaging reports. Studies describing the use of natural language processing, large language models, speech-to-text systems, text summarisation tools, or multimodal approaches combining imaging and textual data were also considered. No language restrictions were applied.

Studies were excluded if they did not involve the generation of dental reports or clinical documentation, or if AI was not used as part of the report generation process. Articles focusing solely on AI-based diagnostic modelling, image classification, or disease detection without report generation capabilities were excluded. Non-peer-reviewed publications, including editorials, opinion pieces, and conference abstracts without full empirical reports, were also excluded.

### 2.2 Information sources and search strategy

Articles published between January 2015 and December 2025 were reviewed on three databases, namely Embase, MEDLINE via Ovid, and IEEE Xplore, using a set of keywords (Table 2). The search was later updated to include articles up to March 2026.

**Table 2:** Keywords used in literature search.

EMBASE	MEDLINE via Ovid	IEEE Xplore
'dental'/exp OR ‘dental’ OR ‘dentistry'/exp OR ‘dentistry’ OR ‘dental informatics’/exp OR ‘dental informatics'	dental OR dentistry OR dental informatics	dent* OR dental informatics
AND	AND	AND
'natural language processing'/exp OR ‘natural language processing’ OR ‘large language model'/exp OR ‘large language model’ OR ‘data extraction'/exp OR ‘data extraction’ OR ‘data mining'/exp OR ‘data mining’ OR ‘information extraction'/exp OR ‘information extraction’ OR ’summary report'/exp OR ‘summary report'	natural language processing OR large language model OR data extraction OR data mining OR information extraction OR summary report OR text summari$ation	natural language processing OR large language model OR data extraction OR data mining OR information extraction OR text summari$ation
AND	AND	AND
'patient note’ OR ((‘patient'/exp OR patient) AND (‘note'/exp OR note)) OR ‘clinical note’ OR ((‘clinical'/exp OR clinical) AND (‘note'/exp OR note)) OR ‘clinical text’ OR ((‘clinical'/exp OR clinical) AND text) OR ‘medical documentation'/exp OR ‘medical documentation’ OR ‘electronic health record'/exp OR ‘electronic health record’ OR ‘diagnostic imaging'/exp OR ‘diagnostic imaging’ OR ‘radiology report'/exp OR ‘radiology report'	patient note* OR clinical note* OR clinical text* OR medical documentation OR electronic health record OR diagnostic imaging OR radiology report	natural language processing OR large language model OR data extraction OR data mining OR information extraction OR text summari$ation patient note OR clinical note OR clinical text* OR clinical documentation OR electronic health record OR imaging report OR radiology report

**Table 3:** AI in dental report generation.

Study	Type of report generated and features	Process of report generation and AI model used	Model training/dataset	Assessment of dental report	Main outcome/result(s)
Abdaoui et al. 2026 (31)	Dental radiology report based on OPG Report consisted of radiographic findings localised on the radiograph Language: English	Vision transformer (ViT) for patch-based visual feature extraction Text encoding using Bio_ClinicalBERT Report generation using T5-base	700 OPG with corresponding diagnostic reports from the OpenI database	1. Language performance: BLEU, ROUGE, Meteor, CIDEr 2. Clinical efficacy: precision, recall, F1 score 3. Hallucination analysis	1. Language performance: - BLEU-4: 0.865 - ROUGE-L: 0.9203 - METEOR: 0.8688 - CIDEr 5.648 2. Clinical efficacy - Precision: 0.982 - Recall: 0.923 - F1 score: 0.9504 3. Hallucination rate: 2.42%
Balel et al. 2026 (30)	Dental radiology report based on OPG Report consisted of radiographic findings localised on anatomical structures detected on the radiograph Language: English	An Ultralytics YOLOv12 model was trained on annotated radiographs. Structured JSON data were processed by a locally hosted LLM (DeepSeek R1, Llama 3.2, Mistral, SmolLM3, Gemma 3, Qwen3, GPT-OSS) for report generation	30,954 OPG were annotated jointly by two researchers with 14 predefined labels	1. Segmentation model assessed in terms of precision, recall, F1 score 2. 1,050 LLM-generated outputs from 50 OPG were analysed in terms of structural validity, consistency, response latency and output length	1. Segmentation model: - Precision: 0.816 - Recall: 0.626 - F1: 0.708. Highest F1 for ectopic/supernumerary teeth (0.994), impacted teeth (0.990) and implants (0.984). 2. Report generation: - Structural validity: Highest in DeepSeek R1 with lowest hallucination count. - Consistency: highest in Gemma-3 - Response latency: shortest in LlaMA 3.2 (12.04 s) - Average output length: shortest in GPT-OSS (333 tokens) vs. longest in Mistral (502 tokens)
Dasanayaka et al. 2025 (27)	Dental radiology report based on OPG Report consisted of patient history, findings and recommendations Language: English	Blip-2-based image captioning model was used to generate captions for OPG in medical terminology Fine-tuned LlaMA-3-8B model was used for report generation	1,000 OPG image-caption pairs descriptively and 1,200 radiology reports annotated by dental radiology experts Dataset contains both common conditions (caries and restorative work) and uncommon conditions (lesions and jawbone fractures)	Qualitative assessment: weighted average score (between 0 and 10) given by dental professionals for the following items: identification of findings, detail of descriptions, comprehensive reporting, diagnostic accuracy, recommendations clarity and conciseness, medical terminology, ethical considerations. Quantitative assessment: Similarities in radiographic findings between machine translation summary and the reference were assessed with Recall-Oriented Understudy for Gisting Evaluation (ROUGE).	Qualitative: average score for generated captions: 8.1 out of 10. Average score for generated radiology reports: 7.5 out of 10. Quantitative assessment (ROUGE): detection accuracy above 80% for common radiographic findings including dental caries (87.9%), impacted teeth (89.7%), bone loss (88%) and periapical lesions (81.8%); detection accuracy was lower for bone fractures (60%) and orthodontic issues (62.5%)*.*
Gao et al. 2024 (28)	Dental radiology report based on OPG Report consisted of radiographic findings localised on anatomical structures detected on the radiograph Language: Chinese	Multi-Level objective Alignment Transformer (MLAT) network, consisting of Holistic-Level Encoder (HLE), Object-Level Collaborative Encoder (OLCE) (used for positional alignment) and Hierarchical Constraint Generator (HCG), which utilises GPT2-Chinese to generate the final report	562 sets of OPGs and their reports, annotated by 13 professionals. Training set: 450 (80.07%) image-report pairs Testing set: 112 (19.93%) image-report pairs Radiographic images OPG of permanent teeth only; reports written by experienced dentists	BLEU-1 to BLEU-4, Meteor, Rouge-L and BERTScore.	MLAT, an object-based approach, can generate reports with correct diagnosis, describe its location, and severity of disease, and outperforms holistic-based methods. Results of metrics: BLEU-1: 50.11 BLEU-2: 35.71 BLEU-3: 27.19 BLEU-4: 21.69 Meteor: 26.34 Rouge-L: 51.30 BERTScore: 0.7813
Stephan et al. 2024 (2)	Dental radiology report based on OPG Report consisted of radiographic findings listed out on a checklist Language: German	Dental radiology reports were generated by ChatGPT 4.0 based on a clinician checklist. This was compared to reports manually drafted by dental students.	100 manually drafted reports and 100 checklists were completed by 100 dental students on 2 different OPGs	1. Readability: Flesch reading ease (FRE) score and Lesbarkeitsindex (readability index; LIX) 2. Text similarity (AI vs. student): BERTScore. 3. Text accuracy: number of included findings vs. contained in the referring checkbox list 4. Descriptive text analysis: measurement of word count, sentence length, syllable count, diphthong count, and character count	1. Readability (FRE score): - AI-generated reports: mean 50.55 (SD 7.80) - student-written reports: mean 51.19 (SD 5.02) readability (LIX index): - AI-generated: mean 48.98 (SD 5.0) - student-written: mean 48.0 (SD 2.85) 2. Text similarity (BERTScore): - precision: mean 0.967 (SD 0.037) - recall: mean 0.958 (SD 0.037) - F_1_ score: mean 0.962 (SD 0.036) 3. No. of findings: - AI-generated reports: mean 41.3 (SD 6.0) - student-written reports: mean 44.6 (SD 7.0) 4. Word count: - AI-generated reports: mean 265.6 (SD 95.4) - student-written reports: mean 200.6 (SD 37.3) Reduction in sentence length (*p* = 0.007), syllables (*p* < 0.01), diphthongs (*p* < 0.01) and characters (*p* < 0.01) in AI-generated reports
Stephan et al. 2025 (25)	Dental radiology report based on OPG (an extension of Stephan et al. 2024) Language: German	Each of the 100 original AI-generated texts was reformulated into a simplified and an accessibility-optimised versions for patients using ChatGPT (GPT-4). Three groups of patients (*n* = 100 in each group) evaluated one type of report each (original, simplified, accessibility-optimised)	AI-generated reports from the 2024 study	1. Sentence count, word count and proportion of long words 2. Readability: FRE score and LIX index 3. Patient questionnaires for clarity, tone, structure and patient engagement	1. Sentence count (*p* < 0.001): - Original: mean 15.2 (SD 3.1) - Simplified: mean 17.2 (SD 3.2) - Accessibility-optimised: mean 27.5 (SD 3.0) Word count: - Original: mean 200.6 (SD 37.3) - Simplified: mean 302.6 (SD 43.3) - Accessibility-optimised: mean 306.1 (SD 43.8) Proportion of long words: accessibility-optimised < simplified version < original (*p* < 0.001) 2. Readability (FRE score) (*p* < 0.001): - Original: 51.1 - Simplified: 55.0 - Accessibility-optimised version: 56.4 Readability (LIX index) (*p* < 0.001) 3. Patient questionnaires indicated higher ratings for the simplified and accessibility-optimised texts for clarity (*p* < 0.001), tone (*p* < 0.001), structure, and patient engagement.
Zhang et al. 2021 (32)	Clinical report based on dental charting Language: English	Audio recording of 20 simulated oral examinations was manually transcribed for model training. An NLP algorithm written in JAVA which contained common linguistic rules and pseudocode was used to analyse audio transcripts.	Four case vignettes, derived from patients 3–12 years, were used for simulated oral examination performed by residents or year 4/5 dental students.	Validation of algorithm and obtaining 1. mean recall (sensitivity over true positive) and 2. precision based on case vignette (ground truth)	1. Recall: mean 99.0% (SD 3.3%) 2. Precision: mean 97.8% (SD 4.1%) with no statistical difference from human charting.

OPG, orthopantomogram; ROUGE, reference-based overlap utility for gisting evaluation; CIDEr, consensus-based image description evaluation; LlaMA, large language model meta AI; NLP, natural language processing; SD, standard deviation.

### 2.3 Study selection and data extraction

Search results were uploaded to the systematic review platform Rayyan (26). Following duplicate removal, MY and EN independently reviewed the titles and abstracts. Full texts of potentially relevant studies were retrieved and assessed against the eligibility criteria. If disagreement occurred, this was resolved through discussion and consensus with a third reviewer (MB).

A data extraction form was developed to extract data on study details, including the country of origin, funder, type of AI model used, specialty/domain of the study, type of data (textual/imaging), language of the data/report, intended audience of the report, main findings and source(s) of funding. The results were presented in tabular and narrative formats.

## 3 Results

### 3.1 Selection of sources of evidence

**Figure 1:**
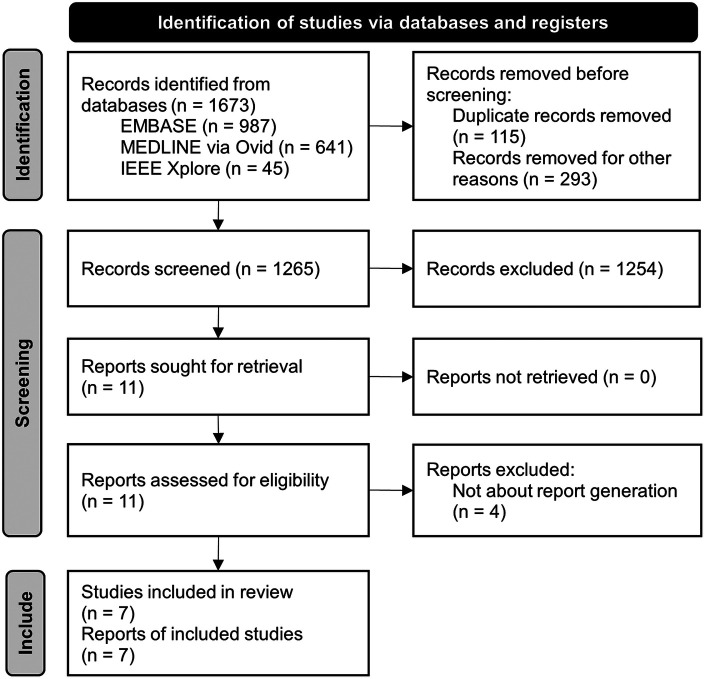
Flowchart of selection of articles.

A total of 1,265 records were identified in the literature search of all databases after removal of duplicates. After reviewing the titles and abstracts for relevance, 11 full text articles were retrieved. Finally, 7 studies were included in the review (Figure 1).

### 3.2 Study characteristics

The characteristics of included studies are presented in Table 3. Of the 7 articles, one study was conducted in Sri Lanka (27), one in China (28), two in Germany (2, 29), one in Turkey (30), one in Tunisia (31), and one in the United States (32). Six studies generated dental radiology reports from radiographs (specifically panoramic views/orthopantomograms=OPG) (2, 27–31), while one reported intraoral dental examination results in a clinical report with dental charting (32).

In terms of method of data collection, three studies used pre-existing image and caption/diagnostic report datasets from OPG (27, 28, 31), of which two studies did not specify the use of only OPG containing permanent dentition (27, 31). One study involved expert annotation of OPG using annotation software (30). One study utilised spreadsheets transcribed from manually completed OPG imaging checklists as training data (2), and the generated dental radiology reports formed the basis for textual accessibility transformation in a follow-up study (29). One study collected transcriptions of simulated oral examinations performed by dental students, which were prepared from paediatric case vignettes (32).

In terms of end-users, while two studies involved reports specifically drafted as patient-facing documents (2, 29), all other studies generated reports only intended for expert users (dental professionals) (27, 28, 30–32). Notably, the study by Stephan et al. explicitly evaluated post-generation adaptation of report text to improve accessibility for patients with varying levels of health literacy (29).

The language of the final generated reports included two in German (2, 29), four in English (27, 30–32) and one in Chinese (28).

### 3.3 Data collection methods and artificial intelligence model

The content included in the final generated report could be either collated manually or facilitated with AI, as part of the report generation algorithm. In the six studies using dental radiographic imaging as a source for reporting, three methods were used to obtain information for report generation: The first method involved using an AI algorithm to directly identify and/or locate findings in a process called ‘image captioning’ (27, 28). A similar approach using visual encoding prepares an input image to facilitate cross-modal alignment with clinical text to localise radiographic findings on the image (31). The second method relied on a checklist of radiographic findings prepared by human specialists (2, 29) before being used to generate the final report. Balel and coworkers used a different method involved clinician annotation using software which is directly used for model training with an object detection system (30). In the first strategy, image-caption or image-report pairs arising from dental radiology reports were used as training dataset to enable the algorithm to identify and/or locate findings in new radiographs (27, 28, 31), whereas in the second strategy, findings were identified by clinician on a pre-designed checklist which was directly fed into a report generation algorithm (2). The third strategy involved labelling teeth and radiographic findings with bounding boxes or polygonal annotation methods using a dedicated annotation software, which is directly used for model training (30). In a final example, the contents of dental reports were generated directly from a narrated clinical examination on four case vignettes, where the narrated audio was processed by an NLP algorithm (32).

### 3.4 Dental report generation

The final dental report is a textual description of clinical/radiographic findings and gives clear and specific recommendations whenever necessary.

All included studies adopted a generalist approach, with no AI systems specifically tailored to individual dental specialties. In studies generating reports from panoramic radiographs, model outputs were limited to commonly encountered radiographic findings, such as caries, restorations, cysts, and other lesions (27, 28, 30, 31).

In two studies, report content was highly standardised, as generation was constrained by pre-designed diagnostic checklists, i.e., adopting a template-based method, thereby limiting the inclusion of unanticipated or incidental findings (2, 29). Similarly, the study based on intraoral dental examination data was restricted to predefined dental charting categories, capturing findings such as caries and restorations but excluding other clinical anomalies outside the charting framework (32).

In all included studies, natural language processing (NLP) algorithms were used for generating the final text based on clinical/radiographic findings. This included the publicly available GPT2-Chinese (28), ChatGPT 4.0 (2, 29), LlaMA-3-8B (27) and T5-base (31). One study compared several NLP models, including ChatGPT 5.0, GPT-OSS, LlaMA 3.2, DeepSeek R1, Gemma-3, Qwen-3, SmolLM3 and Mistral (30). One research team developed their own NLP coded in JAVA computer language (32).

### 3.5 Assessment of the generated dental report

Several metrics were used as quantitative assessment of the final generated report in terms of 1) quality of summary, including accuracy and semantic similarity, and 2) readability, such as word or sentence length.

For the quality of summary, three main metrics, including the Recall-Oriented Understudy for Gisting Evaluation (ROUGE) metric, the Bidirectional Encoder Representations from Transformers (BERT) score and the Bilingual Evaluation Understudy (BLEU) metric were used (27, 28). In each case, quality of the final generated summary was compared against a human reference often by tokenising the sentences involved and comparing the overlap of the candidate (i.e., the generated text) and the reference (i.e., human reference), aiming to have as close to human judgement as possible (33). Alternatively, Balel and co-workers compared the quality of summary generated by different LLM with five objective parameters including structural validity (percentage of correctly parsed outputs), consistency (similarity across repeated responses for identical inputs), response latency and output length in terms of token count (30). In addition to the common metrics used for language performance, one study incorporated hallucination analysis, which involved automatic identification of new content in the generated report that were absent in the reference reports (31). As for the study generating reports using dental charting, direct comparison of items included in the final report with the validation transcripts and case vignettes (ground truth) provided a straightforward calculation for content accuracy (32). Although individual studies have indicated the feasibility of their final model in a clinical situation and commented favourably about their end product based on assessment metrics, it is currently not possible to compare different algorithms directly due to heterogeneity of tasks in the included articles.

In terms of readability, Stephan and co-workers compared simplified and accessibility-optimised versions of reports with the original AI-generated report by measuring word and sentence length, syllable, diphthong and character count (29). As their reports were drafted in German, two validated scores, the Flesch reading ease (FRE) score and the Lesbarkeitsindex (= readability index; LIX), were used to quantify the level of readability of each report version. Although this could demonstrate the capability of AI to customise reports for the intended readership, it was not directly possible to compare report generation in different languages due to variations in language structures, such as word length and order.

Alternatively, qualitative assessment of the generated report was carried out using weighted average scores provided by dentists with different levels of experience, where scores provided by dentists with over ten years of experience were given a higher weighting than scores given by a recent graduate (27). In the study performed by Stephan and coworkers, a structured patient questionnaire was designed to assess clarity, structure and information content, empathy and tone, guidance and motivation for action, and future perspective (29).

### 3.6 System level aspects of dental report generation

With respect to interoperability, there was limited evidence of integration either between AI components or with existing clinical information systems. In five studies, image analysis or automated data extraction modules were linked to downstream report generation pipelines (27, 28, 30–32), whereas the other two relied entirely on manual input of clinical or radiographic findings without connection to existing digital systems (2, 29). Model interchangeability or sharing of datasets were not mentioned in the studies included.

Human-in-the-loop setup (HITL) was not observed in all included studies. Although most studies appeared to involve humans in the initial preparation of training data (such as annotation of dental radiographs (27, 28, 30) and simulated dental clinical examination on case vignettes (32)), none of the studies reflected a true HITL setup, since humans were only involved in the data collection but there was no human clinical feedback or supervision in the model training or report generation.

## 4 Discussion

### 4.1 Current approaches to AI-driven dental report generation

The seven studies included in this scoping review demonstrated the emergence of diverse AI algorithms designed or adapted for dental report generation. Large language models (LLMs) were trained to produce various types of dental reports using heterogeneous input modalities, including radiographic images, transcriptions of clinical examination, and clinician-completed checklists of findings. Some systems also incorporated post-processing adjustments to modify language complexity, thereby improving accessibility for different audiences. The generated outputs included both radiology reports and clinical examination reports, with certain systems integrating speech recognition and real-time dictation functionalities.

The types of dental reports generated included both radiology reports and clinical dental examination reports through voice recognition and dictation. Across the included studies, generated reports were evaluated using multiple assessment approaches, including validated readability indices, clinician-rated content accuracy, and patient feedback questionnaires. Iterative model refinement generally resulted in improved performance, with later versions receiving more favourable ratings from both clinicians and patients. These findings suggest preliminary feasibility and the potential for AI systems to generate dental reports of reasonable quality. However, meaningful comparison between studies was limited due to heterogeneity in methodologies, evaluation metrics, report types, and model architectures.

Overall, while the included studies demonstrated encouraging progress in automated dental report generation, the extent to which this task has been fully resolved remains limited. Most existing approaches focused on narrow, well-defined tasks, such as radiographic captioning or structured data summarisation, often evaluated using retrospective datasets or controlled experimental conditions. Relatively few studies reported prospective validation, external testing, or deployment within real-world clinical workflows. Furthermore, challenges related to generalisability across diverse patient populations, imaging modalities, and clinical settings remain insufficiently addressed. As such, current evidence suggests that automated dental report generation is still an emerging capability, with most systems positioned at the level of proof-of-concept or early-stage development rather than mature, clinically integrated solutions.

The studies collectively demonstrate the viability of free-text generation in dental reporting. Nevertheless, structured dental reporting frameworks may further enhance documentation quality by ensuring that all relevant attributes and clinical domains are systematically addressed (34). Comparable technologies have already been explored in recent commercial AI applications for medical and dental note-taking, i.e., ‘scribe’ programmes, where structured and automated note generation occurs in real time at chairside (35). Such systems may serve as a conceptual bridge between free-text generative models and more standardised reporting formats.

In addition to text generation, some AI-driven report systems incorporated image captioning and automated data extraction, whereby findings were identified algorithmically rather than manually entered prior to report generation (28, 30, 31). This integration of computer vision with generative language models represents a significant advancement toward multimodal dental documentation.

Although limitations in image detection accuracy were highlighted in imaging-focused studies (28, 32), these concerns primarily related to the initial data extraction phase rather than the linguistic generation of the report itself. This suggests a persisting technological gap between reliable computer vision outputs and fully autonomous report generation based on dental images. In practice, clinician verification, potentially through structured checklists, may remain necessary to ensure diagnostic reliability (2). Additionally, there appears to be a paucity of AI systems capable of generating comprehensive and clinically appropriate treatment recommendations based on examination findings and imaging data. Treatment planning involves complex, multi-factorial decision-making processes that extend beyond language generation and require higher-level reasoning capabilities.

When assessing the quality of dental reports, included studies primarily evaluated report accuracy, completeness, readability, and patient perception using diverse scoring systems and validation metrics. However, patient feedback focused largely on perceived ease of language comprehension rather than objective understanding. Patients may report that a document is easy to read while still misunderstanding its clinical implications (36). Over-simplification in the pursuit of accessibility may also result in omission of essential clinical details. Future evaluations should therefore incorporate objective measures of patient comprehension to determine whether AI-generated reports effectively communicate accurate and meaningful information.

While AI-assisted report generation has the potential to streamline documentation processes and reduce administrative burden, it is important to recognise that such systems fundamentally lack autonomous clinical reasoning and remain dependent on the quality and scope of their training data (37). AI-generated reports may serve as supportive tools, particularly for less experienced clinicians, but ethical implementation requires that human clinicians retain ultimate responsibility for verifying accuracy, ensuring completeness, and preventing errors (38). Nevertheless, well-designed and rigorously trained AI systems using high-quality datasets may meaningfully enhance the efficiency and standardisation of dental documentation.

### 4.2 Challenges in interoperability

Improving interoperability of AI systems for report generation could enable safe and scalable deployment of AI-driven dental report generation systems as this allows oral health data across modalities and systems to be integrated and support continuity and safety of care (39).

Despite its importance, interoperability remains a largely unresolved challenge in automated dental report generation. Among the studies included in this review, only five implemented multiple AI components concurrently, generally combining image analysis with downstream report generation and some degree of automatic data extraction (27, 28, 30–32). In contrast, the remaining studies focused primarily on text generation, assuming that clinical inputs were provided manually and without demonstrating integration with real-world clinical data systems (2, 29). Although several studies employed large language models (LLMs) for report generation, there was minimal discussion of how these implementations integrated multiple data modalities—such as radiographic images, structured odontograms, and clinical findings—or how information was exchanged, harmonised, and aligned across modalities to generate a single, unified report. This might limit their readiness for integration into interoperable clinical systems such as electronic dental records.

Even within a single data modality, such as clinical text, interoperability challenges persist. The adoption of harmonised clinical coding systems (such as SNOMED CT) may improve semantic interoperability by enabling consistent data classification and exchange (40). However, multimodal integration may still be required within the same modality to account for different clinical contexts, such as chief complaints vs. physical examination findings. Future work may therefore benefit from hybrid or protocol-based approaches to context integration, or from emerging cross-platform frameworks such as MedScreenDental, which aim to support structured, context-aware dental data integration across AI systems (41).

Although this review focused on dental applications of AI in report generation, it reiterates findings from the medical literature using LLM in clinical text summarisation, such as drafting of clinical letters (21), preparation of discharge summaries (20), fine tuning of algorithms to customise specific attributes such as report length and language complexity (3), use of voice input technologies (42) and assessment of the algorithm and resultant text (43). The uniqueness of dentistry, being largely focused on intraoral findings and unique procedures such as restorative and rehabilitative work, necessitates AI models specialised and fine-tuned to meet the need for dental text summarisation and report generation.

### 4.3 Limitations

The inclusion of only seven articles in our review suggests that dental report generation remains a relatively underexplored and technically demanding area. Although commercial products have emerged which automates dental documentation within clinical reporting workflows, peer reviewed evidence of these systems remain scarce. This likely reflects both a genuine gap in research interest and the inherent challenges associated with studying this specific topic.

Several factors may account for this scarcity of literature. First, although there has been a surge in AI studies focusing on dental feature extraction, for example computer vision for caries detection or bone loss assessment and data-driven diagnostic support like EHR-based periodontal classification, relatively few studies have bridged the gap toward natural language generation and automated clinical reporting. Current research efforts are therefore predominantly concentrated on upstream analytical tasks rather than downstream communication and documentation processes.

Second, developing AI systems for report generation is inherently more complex, as it requires not only accurate data interpretation but also coherent and contextually appropriate language generation. This dual requirement combining clinical reasoning with linguistic fluency introduces additional methodological and technical challenges compared to purely diagnostic models.

Third, report generation is highly language-dependent. Models trained in one language are not directly transferable to another due to differences in syntax, grammar, and clinical terminology. Moreover, region-specific datasets often vary in population characteristics and documentation styles, necessitating localised model development and limiting scalability.

Finally, barriers related to data availability and governance may further hinder research in this area. Clinical documentation datasets are frequently sensitive, unstructured, and difficult to standardise, restricting access for model training and external validation. Expansion of foreign language corpus would facilitate the development of AI report generation tools in their respective languages (44).

Collectively, these factors help explain the relatively slow pace of research development and publication in automated dental report generation, highlighting a critical area for future investigation.

The reviewed literature revealed several key limitations. First, medical terminology and synonymous expressions may be treated as distinct entities by AI systems, potentially resulting in redundant, repetitive, or inaccurate reporting where identical findings are described multiple times (28, 32, 45). Second, rare conditions may be insufficiently represented in training datasets, limiting model performance in atypical cases. This issue is exacerbated in languages with relatively small available corpora for model training (27). Furthermore, small or unbalanced datasets may reduce algorithm robustness, particularly in handling ungrammatical input or typographical errors (45). Proposed mitigation strategies include expanding training datasets and developing more comprehensive language corpora to better capture the nuances of medical and dental terminology across languages.

Transferability across languages presents additional challenges. AI report generation systems are not directly adaptable between languages, particularly those from different language families, due to variations in grammar, syntax, sentence structure, abbreviations, and professional conventions. Similarly, evaluation of AI-generated reports is not directly comparable when report types differ fundamentally, such as imaging reports vs. clinical examination summaries. Comparisons of content accuracy may be more meaningful when confined to similar report categories—for example, between clinical examination reports containing intraoral findings and special tests, or between procedural reports detailing treatment techniques and materials used. Moreover, reports generated from voice dictation should be evaluated differently from those derived from structured written text or imaging data, as the underlying algorithmic processes differ substantially.

### 4.4 Implications for clinical practice

From a clinical perspective, AI-assisted report generation may improve efficiency, reduce administrative burden, and support more consistent and comprehensive documentation, ultimately contributing to improved quality of care and communication among dental professionals and with patients.

Dentistry is inherently multimodal, involving textual patient histories, structured clinical examinations, radiographs, intraoral photographs, and 3D imaging. Therefore, a single AI model is unlikely to perform optimally across all these data types and specialties. Model selection should therefore be guided by the specific data characteristics and reporting priorities of each specialty. For example, endodontics may require high-resolution imaging analysis to evaluate pulp and canal morphology, while periodontology may rely more on structured clinical measurements and longitudinal EHR data. Modular architectures enable such specialty-specific customisation within a unified framework, allowing models to be tailored to particular tasks without redesigning the entire system, thereby enhancing both scalability and clinical relevance.

### 4.5 Implications for future research

Currently, studies are often conducted in well-controlled or restricted cohorts, which may limit the generalisability of developed models to broader patient populations and clinical settings despite demonstrating high performance metrics. Future study designs should incorporate multi-centre and multi-institutional datasets to better capture diverse population characteristics and technical variability, such as differences in radiographic systems and acquisition protocols.

To bridge the gap between data extraction and clinical reporting, future research should focus on developing integrated, end-to-end frameworks that directly translate analysed data into structured clinical reports and summaries. Such frameworks should aim to combine multiple data modalities, including clinical text, imaging, and electronic health records, to reflect real-world clinical workflows.

In addition, future studies should adopt standardised evaluation metrics and reporting frameworks to enable meaningful comparison across studies and facilitate external validation. In terms of language requirements, the development of multilingual and domain-specific medical corpora will be important to support robust and generalisable report generation using large language models. Prospective validation in real-world clinical environments and assessment of clinical utility, user acceptability, and workflow integration will be essential to support translation into routine practice.

AI models trained to meet, or even exceed, established reporting criteria may serve as valuable reference tools for both learners and clinicians. The interactive nature of these AI algorithms can support self-directed and adaptive learning, allowing students to compare AI-generated outputs with their own interpretations when processing raw clinical data and findings, such as dental charting, case synopses, and photographic/radiographic imaging.

In addition, these tools may help standardise clinical documentation practices by reinforcing established reporting guidelines and improving consistency in clinical communication. With appropriately fine-tuned models, students can repeatedly practise analysing and interacting with real or simulated clinical cases at their own pace, thereby enhancing clinical reasoning, diagnostic interpretation, and documentation skills.

Finally, AI-assisted report generation should be regarded as a supportive documentation tool rather than a replacement for clinical judgement. Future research should prioritise larger multilingual datasets, standardised evaluation criteria, structured reporting integration, and objective measures of patient understanding. Addressing these gaps will be essential to ensure that AI-driven dental reporting systems are safe, reliable, ethically implemented, and capable of meaningfully enhancing clinical workflows and patient communication.

## 5 Conclusion

This scoping review highlighted the emerging role of artificial intelligence in dental report generation, demonstrating early feasibility across multiple input modalities, including imaging, structured data, and voice transcription. The effectiveness of such AI tools was evaluated using various summary or machine translation assessment tools as well as clinician/patient feedback, but comparisons among models were not possible due to heterogeneity of report types. Although current systems show promise in generating reasonably accurate and readable reports, substantial heterogeneity in methodologies, evaluation metrics, and report types limits direct comparison and generalisability. Key challenges include linguistic variability, limited dataset diversity, multimodal integration gaps, and the absence of robust evaluation frameworks for patient comprehension and clinical safety.
